# Characterizing Population EEG Dynamics throughout Adulthood

**DOI:** 10.1523/ENEURO.0275-16.2016

**Published:** 2016-12-12

**Authors:** Ali Hashemi, Lou J. Pino, Graeme Moffat, Karen J. Mathewson, Chris Aimone, Patrick J. Bennett, Louis A. Schmidt, Allison B. Sekuler

**Affiliations:** 1 Department of Psychology, Neuroscience, and Behaviour, McMaster University, Hamilton, Ontario, L8S 4K1, Canada; 2 InteraXon Inc., Toronto, Ontario, M5V 1K4, Canada

**Keywords:** age, alpha frequency, Big Data, EEG, mindfulness, Muse

## Abstract

For decades, electroencephalography (EEG) has been a useful tool for investigating the neural mechanisms underlying human psychological processes. However, the amount of time needed to gather EEG data means that most laboratory studies use relatively small sample sizes. Using the Muse, a portable and wireless four-channel EEG headband, we obtained EEG recordings from 6029 subjects 18–88 years in age while they completed a category exemplar task followed by a meditation exercise. Here, we report age-related changes in EEG power at a fine chronological scale for δ, θ, α, and β bands, as well as peak α frequency and α asymmetry measures for both frontal and temporoparietal sites. We found that EEG power changed as a function of age, and that the age-related changes depended on sex and frequency band. We found an overall age-related shift in band power from lower to higher frequencies, especially for females. We also found a gradual, year-by-year slowing of the peak α frequency with increasing age. Finally, our analysis of α asymmetry revealed greater relative right frontal activity. Our results replicate several previous age- and sex-related findings and show how some previously observed changes during childhood extend throughout the lifespan. Unlike previous age-related EEG studies that were limited by sample size and restricted age ranges, our work highlights the advantage of using large, representative samples to address questions about developmental brain changes. We discuss our findings in terms of their relevance to attentional processes and brain-based models of emotional well-being and aging.

## Significance Statement

We collected >6000 participants’ EEG data during two different tasks in uncontrolled environments and identified subtle but robust sex differences in several EEG measures, as well as age-related shifts in EEG activity on a year-by-year scale. Our large sample size provided us with the power to highlight gradual age-related changes in several EEG measures, and how those changes differ between males and females, in a representative population of individuals completing the tasks in uncontrolled, natural environments.

## Introduction

For many decades, electroencephalography (EEG) has been used effectively for different purposes in a variety of fields. For example, clinicians have used EEG to understand several illnesses, including epilepsy and sleep disorders; engineers have used EEG to develop wheelchairs that respond to brain states; and psychologists have used EEG to track the temporal flow of information through the sensory systems and identify neural correlates of psychological processes. Although EEG has been a useful clinical and scientific tool, its applications have been constrained because recording of EEG data is time-consuming and requires laboratories equipped with expensive EEG equipment. Researchers typically collect data from a small sample of participants and hope that other researchers replicate the results to validate inferences about the general population. Using much larger samples would, in most cases, make it easier to establish the robustness and generalizability of empirical findings.

Fortunately, recent technological advances and industry-led innovation have led to the development of research-grade EEG products that are affordable and easily used by consumers. Our focus here is on the Muse, the EEG headband created by InteraXon (Toronto, ON, Canada), who commercialized it as a neurofeedback tool in mindfulness-based stress reduction (MBSR). MBSR-related benefits aside [see Kabat-Zinn (1994) for an explanation of MBSR and Kabat-Zinn (2003) and Davidson et al. (2003) for some empirical evidence of its benefits], arguably the most beneficial aspect of the Muse to researchers has been that the company has amassed hundreds of thousands of sessions of EEG data from tens of thousands of consenting users, making InteraXon, to our knowledge, the holder of the largest EEG database in the world. Not only is the current database valuable and ripe for analysis, the ease of use and low cost of the Muse allows for widespread deployment of the hardware to capture EEG activity outside of the laboratory.

Consumer use of the Muse typically consists of pairing it with a compatible mobile device via Bluetooth technology and using the Muse application to complete a breath-guided meditation session. During each session, users also complete a variation of the Category Exemplar Task which, in combination with the MBSR portion of the session, allows for the EEG to be captured for both a busy mind during the task and a calm mind during the MBSR exercise. The Muse database consists of tagged EEG data representing electrocortical activity recorded at four scalp locations—temporoparietal (TP9 and TP10) and frontal (AF7 and AF8) locations—plus a fifth frontal electrode (Fpz) that is used as the reference, while participants complete the MBSR meditation session and the Category Exemplar Task.

Here, we used the data from thousands of users to study age-related changes in EEG power throughout adulthood. We report several changes as a function of age, including increased power in the α and β bands, an age-related reduction in peak α frequency, and an overall rightward bias in frontal α asymmetry. We discuss the consistency of our findings with previous laboratory studies of attention regulation and other processes thought to be related to mindfulness meditation. We also discuss our findings in the framework of brain-based models of well-being related to aging, as well as the value of Big Data in EEG studies.

## Methods

### Participants

Data were collected from individuals who used the Muse between May 2014 and January 2015 and opted into the optional research program in the accompanying Muse/Calm mobile application. Our original clean database contained 6081 unique users, which then was reduced by excluding users who were <18 years old or who chose not to report their age, for a final count of 6029 individuals. The distribution of the age and sex of the users is displayed in Table 1.

**Table 1.** User and session distribution by age and sex.

Age (years)	Male	Female	Total
18–19	48	17	65
20–29	854	324	1178
30–39	1227	419	1646
40–49	1059	359	1418
50–59	708	344	1052
60–69	400	166	566
70–79	77	20	97
≥80	6	1	7
Total	4379	1650	6029

For each user, data were averaged for up to five sessions.

### Design and procedure

Data were collected using the Muse (formerly known as Calm) mobile application found on the Apple App Store, Google Play, and Amazon Appstore. At the beginning of each user’s first session, the app provided visual and auditory instructions on how to apply the Muse headset to attain optimal signal quality and general information about the Muse application, which provides auditory feedback to assist in MBSR meditation. The auditory feedback resembled the natural sound of wind and ocean waves, with increasing sounds reflecting an active mind, and quietness reflecting a calm mind. The algorithm determining the auditory feedback involved an individual calibration step to establish a baseline. This calibration step was a 1-min phase in which participants completed a version of the Category Exemplar Task: participants were told to close their eyes, and at 0, 20, and 40 s were given a new category for which they were to think of as many examples as they could.

After the calibration (CAL) procedure, the participant began a neurofeedback (NFB) session. The default duration of the NFB session was 3 min, but users could have opted to complete 3-, 5-, 10-, or 20-min sessions. During the NFB session, users were instructed to close their eyes, focus their mind on counting their breaths, silently/mentally acknowledge any deviations of attention from counting their breaths (i.e., mind-wandering), and refocus on counting their breathing. Although this may not be the traditional definition of NFB, we refer to this technique as NFB since the Muse software applies a trade-secret algorithm developed through machine learning to reward a decrease in EEG signatures of mind-wandering.

The amount of data varied significantly across users, with some individuals recording several hundred CAL and NFB sessions. To prevent our analyses from being biased by frequent users, we averaged the first several sessions, up to a maximum of five sessions, to create a single pair of averaged CAL and NFB sessions for each user.

### EEG recording and processing

EEG data were recorded using InteraXon’s Muse headset (RRID:SCR_014418). The Muse is a consumer and research-grade EEG headset with four recording channels (TP9, TP10, AF7, and AF8) referenced to a fifth channel located at Fpz. Active noise suppression was achieved by creating driven right leg (DRL) circuits between two forehead DRL channels and Fpz. The DRL circuits were used to establish that the electrodes have skin contact (i.e., any activity detected by the circuit indicated that the headset was positioned to have skin contact), after which the characteristics of the incoming EEG signal (variance, amplitude, and kurtosis) were used in a decision tree in which low power, low amplitude, and low kurtosis were favored in classifying the real-time signal as clean. EEG was sampled at 220 Hz.

Data were collected from participants from several continents, and the appropriate 50-Hz (Europe and Asia) or 60-Hz (North America) notch filters were applied to each individual session depending on self-reported location. Artifacts were detected by first applying a 2- to 36-Hz bandpass filter on the raw EEG signal. Continuous EEG was then divided into 1.16-s epochs (256 samples), and each epoch’s overall power was compared to a threshold of 275 µV^2^. The threshold was previously determined by large-scale visual inspection to separate clean and noisy data. Only epochs exceeding the threshold were rejected from the EEG session. If >10% of any session at any of the four channels was rejected using this method, then that entire session (NFB and CAL) was excluded from analysis. The database originally contained 139,548 sessions, but applying the rejection criteria above reduced that to 74,321 sessions (i.e., 47% of the sessions were rejected for containing excessive artifacts). We further excluded all sessions beyond the first five clean sessions per user, reducing the database to 22,386 sessions, from 6029 unique users. There were an average of 3.7 sessions per user, with each user having at least one session but no more than five sessions.

### EEG measures

All analyses were done on EEG data from the entire cleaned session. For each session, Matlab’s *fft* function was used compute a power spectrum with a frequency resolution of 1 Hz. Total power (µV^2^) was calculated for the δ (0–2 Hz), θ (3–7 Hz), α (8–13 Hz), and β (14–30 Hz) bands. Lower and upper α power were also quantified in the 8- to 10-Hz and 11- to 13-Hz frequency ranges. Band power was then log_10_-transformed for normalization. Additionally, α asymmetry was calculated by subtracting the log_10_-transformed left α power from the log_10_-transformed right α power separately for the frontal and temporal locations. Finally, α peak frequency, defined as the frequency component in the 8- to 13-Hz range with the highest power, was measured for each person, separately at each channel.

## Results

**Figure 1.**
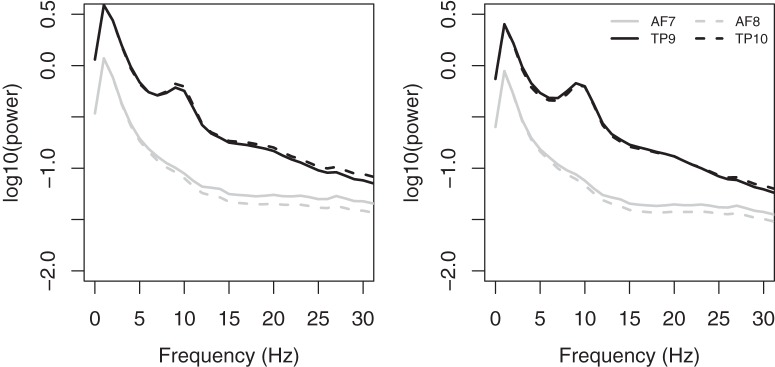
Average power spectra at each channel for CAL (left) and NFB (right) conditions. Frontal and temporoparietal channels are represented by black and gray lines, respectively, and left and right channels in these regions are represented by solid and dashed lines, respectively.

**Figure 2.**
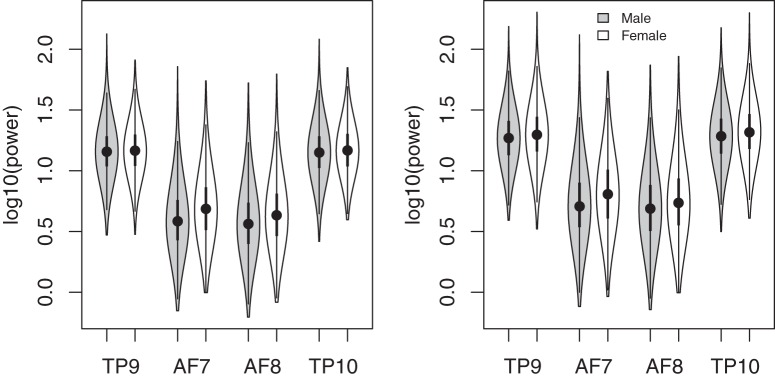
Log_10_-transformed EEG power in the 0- to 30-Hz range measured in females (white) and males (gray) at each channel for NFB (left) and CAL (right), shown in the form of violin plots (Hintze and Nelson, 1998). Filled circles represent the median, and the first and third quartiles are identified by the bottom and top of the bold vertical lines, respectively. The bottom and top of the thin vertical line represent the lower and upper adjacent values, respectively. Females had slightly higher power at all channels, regardless of task.

Power spectra, averaged across users, were calculated separately for the CAL and NFB sessions at each channel (Fig. 1). EEG power was greater in temporoparietal than frontal regions, especially at lower frequencies (Fig. 1). There was also a very noticeable peak in the α frequency range in temporoparietal channels, but not frontal channels. Total power in the 0- to 30-Hz range was significantly higher in females than males at all channels (Fig. 2). For CAL, the sex difference was significant at all channels (*t*_6027_ > 5.08, *p* < 0.00001). For NFB, the sex difference was significant at channels AF7, AF8, and TP10 (*t*_6027_ > 3.4, *p* < 0.001) but not at TP9 (*t*_6027_ = 1.66, *p* < 0.097).

### Band analysis

To evaluate age-related changes in each dependent variable, we used linear models that included age, age^2^, sex, task, and channel, as well as all two-, three-, and four-way interactions, as predictor variables. For all measures, task and channel each had at least one significant interaction with each other, age, age^2^, sex, or the age × sex and age^2^ × sex interaction. Because of these interactions, we proceeded with separate analyses for each channel and task, using linear models that included only age, age^2^, sex, and the age × sex and age^2^ × sex interactions. If either interaction was significant, then separate models that included age and age^2^ as predictors were fitted to data from males and females. Although all analyses were conducted for both CAL and NFB, for brevity, we present the accompanying data figures only from the NFB session. The pattern of results were qualitatively similar across CAL and NFB except in a few cases that we discuss in the text. Furthermore, Table 2presents all of the results from the models fitted to the CAL data. To view the accompanying figures for CAL sessions, please contact the corresponding author.

**Table 2.** Regression coefficients (rounded to nearest 0.00001) estimated for each measure and channel in the CAL condition.

Measure	Channel	Intercept	Age	Age^2^	Sex	Age × sex	Age^2^ × sex	*R*^2^	Age (m)	Age (f)	Age^2^ (m)	Age^2^ (f)
δ	AF7	0.43339^†^	–0.00152***	0.00010***	–0.03076*	0.00028	0.00010	0.210^†^				
δ	AF8	0.42873^†^	–0.00201^†^	0.00013^†^	–0.03468*	0.00101	0.00009	0.280^†^				
δ	TP9	0.95463^†^	–0.00312^†^	0.00011^†^	0.01803	–0.00095	0.00004	0.482^†^				
δ	**TP10**	0.94772^†^	–0.00320^†^	0.00013^†^	0.02609*	–0.00074	0.00001	0.535^†^				
θ	AF7	–0.13675^†^	–0.00064*	0.00007***	0.01798	0.00084	0.00013**	0.380^†^	–0.00064*	0.00021	0.00007***	0.00020^†^
θ	AF8	–0.15823^†^	–0.00095**	0.00008***	–0.00064	0.00091	0.00011*	0.282^†^	–0.00095**	–0.00004	0.00008***	0.00019^†^
θ	TP9	0.43667^†^	–0.00191^†^	0.00006**	0.01132	0.00025	0.00008*	0.393^†^	–0.00191^†^	–0.00166***	0.00006**	0.00013***
θ	**TP10**	0.41830^†^	–0.00225^†^	0.00009^†^	0.03257***	0.00018	0.00005	0.515^†^				
α	**AF7**	–0.32292^†^	0.00090**	0.00006**	0.11225^†^	0.00116*	0.00009*	0.798^†^	0.00090***	0.00206^†^	0.00006***	0.00015^†^
α	**AF8**	–0.36987^†^	0.00071*	0.00007***	0.07154^†^	0.00068	0.00008*	0.645^†^	0.00071*	0.00139**	0.00007***	0.00016^†^
α	TP9	0.45606^†^	–0.00139^†^	–0.00004*	–0.01559	0.00040	0.00015***	0.229^†^	–0.00139***	–0.00099	–0.00004*	0.00011**
α	TP10	0.47902^†^	–0.00145^†^	–0.00000	0.00667	0.00000	0.00012*	0.228^†^	–0.00145***	–0.00144**	–0.00000	0.00011**
β	**AF7**	–0.08567^†^	0.00242^†^	0.00009**	0.26647^†^	0.00072	0.00001	0.846^†^				
β	**AF8**	–0.15873^†^	0.00318^†^	0.00008**	0.20429^†^	–0.00027	–0.00000	0.810^†^				
β	**TP9**	0.34545^†^	0.00173^†^	–0.00005**	0.05945^†^	0.00008	0.00009*	0.608^†^	0.00173^†^	0.00181***	–0.00005**	0.00004
β	**TP10**	0.37780^†^	0.00224^†^	–0.00000	0.07855^†^	–0.00085	0.00007	0.589^†^				
**α Peak**	**AF7**	10.28787^†^	–0.02162^†^	0.00010	–0.00010^†^	0.00122	0.00002	0.510^†^				
α Peak	AF8	10.22824^†^	–0.01597^†^	0.00018	–0.29203***	–0.00341	0.00024	0.351^†^				
α **Peak**	**TP9**	9.57089^†^	–0.01469^†^	0.00002	0.02296	–0.00592*	–0.00010	0.584^†^	–0.01469^†^	–0.02060^†^	0.00002	–0.00008
α **Peak**	**TP10**	9.60727^†^	–0.01367^†^	–0.00004	0.06327	–0.00426	–0.00007	0.567^†^				
α Asym	AF8–AF7	–0.04695^†^	–0.00018	0.00002	–0.04071^†^	–0.00048	–0.00001	0.338^†^				
α Asym	TP10–TP9	0.02296^†^	–0.00006	0.00004^†^	0.02225^†^	–0.00039	–0.00004	0.220^†^				

Bolded rows indicate cases where *R* ^2^ ≥ 0.5. *p < 0.05, **p < 0.01, ***p < 0.001, ^†^*p* < 0.0001.

**Figure 3.**
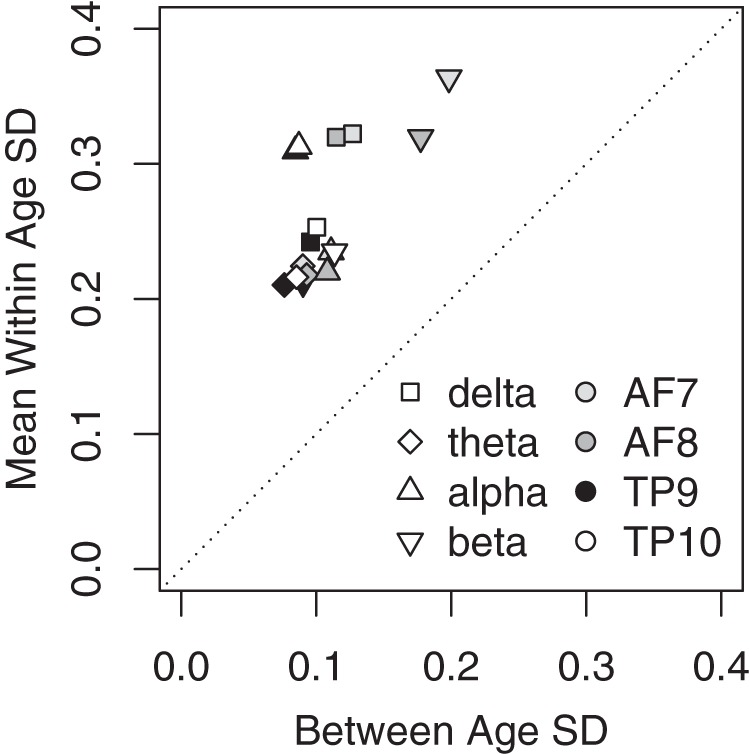
Standard deviation of the average band power across ages (*x*-axis) plotted with the average standard deviation of each band power across participants within each age (*y*-axis). Within-age SD was calculated by calculating the SD across participants at each given age. Ages 78+ all had two or fewer participants, so we grouped them into a single age bin. Mean within-age SD (*y*-axis) was calculated as the average within-age SD. Between-age SD (*x*-axis) was calculated by first computing the mean band power for each individual age, then calculating the SD across these values.

Preliminary analyses indicated that the average within-age variance (i.e., variance across all participants within the same year, averaged across all years) was much larger than the between-age variance, a trend seen across all channels for all measures (Fig. 3). Because we were interested primarily in age-related variance, we used weighted least-squares (WLSs) to fit linear models to the mean at each age, where the weight corresponded to the number of users at each age. This method effectively removes within-subject and within-age variation. The coefficients of the resulting WLS model are identical to a traditional least-squares regression applied to the nonaveraged data from individual users, but the overall fit of the model (i.e., *R* ^2^) is much higher because the averaging removes within-age variance.

In all models, age was treated as an integer variable, and sex (male = 0; female = 1) was a dichotomous variable. Furthermore, to have a more meaningful intercept in our model, age was centered on the mean age of our participants (i.e., 42 years). Therefore, the best-fitting value of the intercept represents the estimate of the dependent variable (e.g., δ power) for males at 42 years of age, the sex parameter represents the difference between males and females at 42 years of age, the age and age^2^ parameters represent the change in the dependent variable that occurs (on average) in males with each unit change in age and age^2^, and the sex × age and sex × age^2^ parameters represent the difference between the age and age^2^ effects in males and females. The results of the regression analyses are shown in Tables 2 and 3. Because of the large sample size, the linear model accounted for a statistically significant portion of the variance in every case. However, for the sake of brevity, our discussion focuses on the subset of cases in which the linear model accounted for at least 50% of the age-related variance.

**Table 3.** Regression coefficients (rounded to nearest 0.00001) estimated for each measure and channel in the NFB condition.

Measure	Channel	Intercept	Age	Age^2^	Sex	Age × sex	Age^2^ × sex	*R* ^2^	Age (m)	Age (f)	Age^2^ (m)	Age^2^ (f)
δ	AF7	0.29099^†^	–0.00202^†^	0.00013^†^	–0.02249	0.00133	0.00011	0.267^†^				
δ	AF8	0.28779^†^	–0.00267^†^	0.00013^†^	–0.02768	0.00130	0.00012*	0.319^†^	–0.00267^†^	–0.00137	0.00013^†^	0.00025^†^
δ	**TP9**	0.76347^†^	–0.00388^†^	0.00011^†^	–0.00821	–0.00021	0.00006	0.620^†^				
δ	**TP10**	0.74835^†^	–0.00418^†^	0.00014^†^	0.00111	0.00013	–0.00000	0.662^†^				
θ	AF7	–0.24320^†^	–0.00136^†^	0.00008^†^	0.02290*	0.00207***	0.00010*	0.412^†^	–0.00136^†^	0.00071	0.00008***	0.00019^†^
θ	AF8	–0.26573^†^	–0.00146^†^	0.00007***	0.00793	0.00161**	0.00013**	0.365^†^	–0.00146^†^	0.00015	0.00007**	0.00021^†^
θ	TP9	0.35367^†^	–0.00196^†^	0.00004*	–0.02996**	0.00082	0.00014***	0.373^†^	–0.00196^†^	–0.00113*	0.00004*	0.00017^†^
θ	TP10	0.31149^†^	–0.00262^†^	0.00009^†^	–0.00430	0.00087	0.00010*	0.480^†^	–0.00262^†^	–0.00175***	0.00009^†^	0.00018^†^
α	**AF7**	–0.39869^†^	0.00123^†^	0.00007***	0.11474^†^	0.00206***	0.00008*	0.815^†^	0.00123^†^	0.00329^†^	0.00007***	0.00015^†^
α	**AF8**	–0.44195^†^	0.00101**	0.00006***	0.08223^†^	0.00142*	0.00010*	0.712^†^	0.00101**	0.00243^†^	0.00006**	0.00016^†^
α	TP9	0.48919^†^	0.00049	–0.00005*	–0.02840*	0.00065	0.00016**	0.105**	0.00049	0.00114	–0.00005*	0.00011**
α	TP10	0.47046^†^	0.00024	–0.00002	–0.00498	0.00052	0.00013**	0.087**	0.00024	0.00075	–0.00002	0.00012**
β	**AF7**	–0.18422^†^	0.00197^†^	0.00011***	0.26647^†^	0.00174*	0.00004	0.868^†^	0.00197^†^	0.00372^†^	0.00011^†^	0.00015**
β	**AF8**	–0.23850^†^	0.00233^†^	0.00008**	0.21553^†^	0.00046	0.00003	0.813^†^				
β	**TP9**	0.29314^†^	0.00206^†^	–0.00005**	0.05542^†^	0.00067	0.00012**	0.669^†^	0.00206^†^	0.00273^†^	–0.00005**	0.00006*
β	**TP10**	0.28957^†^	0.00216^†^	0.00000	0.08575^†^	0.00011	0.00007	0.675^†^				
**α Peak**	**AF7**	9.72796^†^	–0.03847^†^	0.00052***	–0.03651	0.00159	–0.00028	0.779^†^				
**α Peak**	**AF8**	9.82156^†^	–0.03457^†^	0.00010	–0.05259	–0.00074	–0.00009	0.665^†^				
α **Peak**	**TP9**	9.46783^†^	–0.01795^†^	0.00001	0.11219*	–0.00647*	–0.00032	0.723^†^	–0.01795^†^	–0.02442^†^	0.00001	–0.00030
α **Peak**	**TP10**	9.54145^†^	–0.01891^†^	–0.00000	0.06429	–0.00865***	0.00004	0.768^†^	–0.01891^†^	–0.02756^†^	0.00000	0.00004
α Asym	AF8–AF7	–0.04326^†^	–0.00022	–0.00000	–0.03251^†^	–0.00064	0.00002	0.235^†^				
α Asym	TP10–TP9	–0.01873^†^	–0.00025	0.00004^†^	0.02342^†^	–0.00013	–0.00003	0.246^†^				

Bolded rows indicate cases where *R*^2^ ≥ 0.5. *significance levels:* *p < 0.05, **p < 0.01, ***p < 0.001, ^†^*p* < 0.0001.

#### Delta power

**Figure 4.**
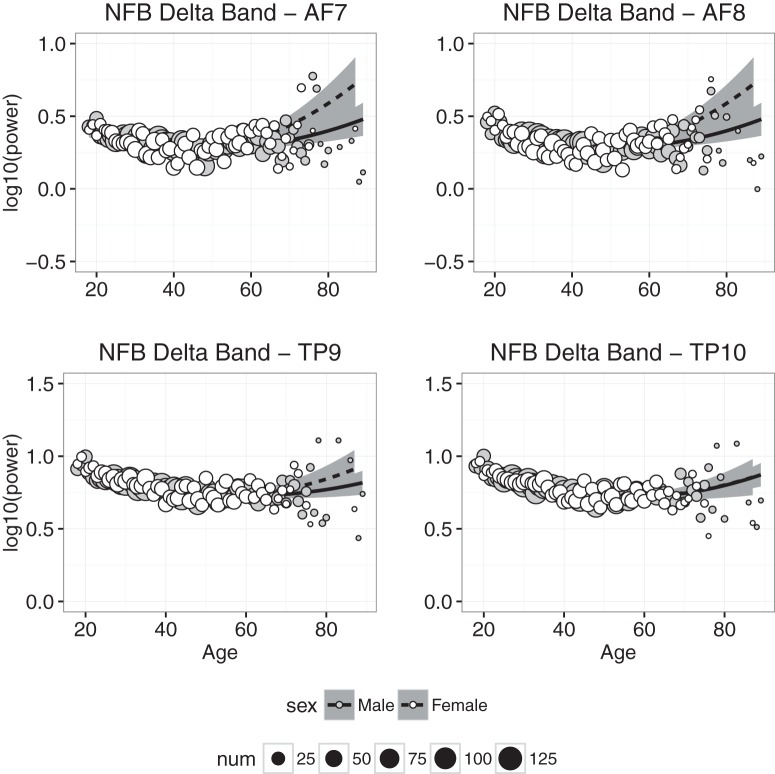
Delta band power plotted against age for males (gray symbols) and females (white symbols). Each point represents the mean for that age; symbol size represents how many individuals were used to compute the mean. Regression was used to compute the best-fitting curves separately for males (solid line) and females (dashed line), and the shaded regions represent 95% confidence intervals.

Delta power measured at each electrode in the NFB condition is plotted as a function of age in Fig. 4, and the results of the regression analyses in the CAL and NFB conditions are shown in Tables 2 and 3. In the CAL condition, the regression model accounted for statistically significant amounts of age-related variance at all electrodes, but accounted for ≥50% of age-related variance only in channel TP10 (and 48% of the variance in TP9). Similar results were obtained in the NFB condition: all of the fits accounted for statistically significant amounts of variance, but accounted for ≥50% of the variance only in the two temporoparietal channels. In both conditions, δ power decreased between 20 and 40 years of age, and then leveled off or increased slightly beyond ≈50 years of age. We also found that, in the CAL condition, the effect of sex differed significantly from zero (TP10 β = 0.02609, *p* < 0.02), suggesting that δ power was slightly higher in females than males.

#### Theta power

**Figure 5.**
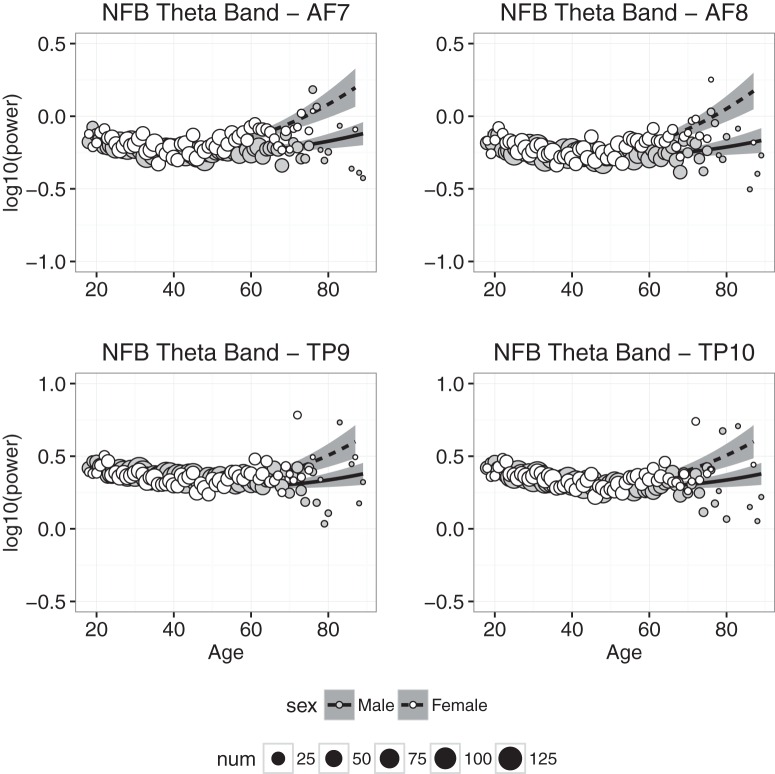
Theta band power plotted against age for males (gray symbols) and females (white symbols). Each point represents the mean for that age; symbol size represents how many individuals were used to compute the mean. Regression was used to compute the best-fitting curves separately for males and females, and the shaded regions represent 95% confidence intervals.

Theta power measured at each electrode in the NFB condition is plotted as a function of age in Fig. 5. The figures indicate that the effects of age on θ power were qualitatively similar to those found with δ power. For example, as was the case with δ power, θ power decreased slightly between 20 and 40 years of age and increased slightly beyond 50 years of age. There also is an indication that sex differences were larger in individuals older than 60 years of age. However, a comparison of Figs. 4 and 5 suggests that age-related changes in θ were smaller than age-related changes in δ. The regression results are consistent with these observations: as was found with δ power, only the regression on data from the temporoparietal channel TP10 accounted for large amounts (i.e., ≈50%) of age-related variance in θ power, and the significant effect of sex at TP10 and the significant interactions between sex and either age or age^2^ in almost all cases reflected greater θ power for females than males, especially at later years. However, the best-fitting coefficients for the age and age^2^ variables were smaller for θ power than for δ power.

#### Alpha power

**Figure 6.**
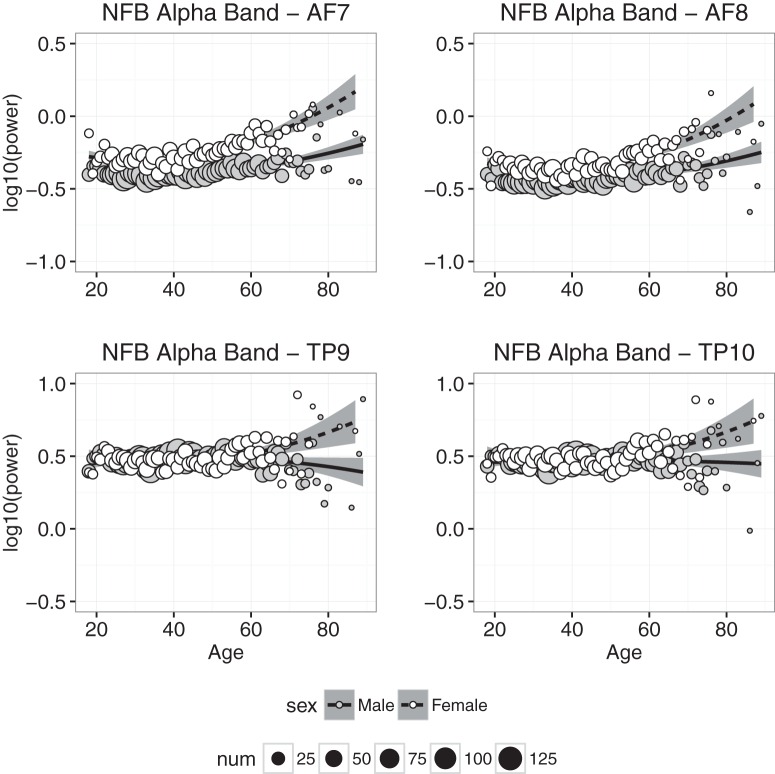
Alpha band power plotted against age for males (gray symbols) and females (white symbols). Each point represents the mean for that age; symbol size represents how many individuals were used to compute the mean. Regression was used to compute the best-fitting curves separately for males and females, and the shaded regions represent 95% confidence intervals.

Alpha power measured at each electrode in the NFB condition is plotted as a function of age in Fig. 6. A comparison of Fig. 6 to Figs. 4 and 5 suggests that age-related changes in α power differed from age-related changes in δ and θ power. For example, sex differences in α power, particularly at frontal electrodes, are much larger than those observed for δ and θ power. Also, unlike what was found with δ and θ power, age-related changes in α power appear to be greater at frontal than temporoparietal electrodes, and furthermore α power appears to increase, not decrease, with age. The regression analyses were consistent with these observations. In the CAL and NFB conditions, the linear models accounted for significant portions of age-related variance at all electrodes, but for at least 50% of age-related variance only at AF7 and AF8. Also, the best-fitting coefficient for age was positive at frontal sites, indicating that α power, unlike δ and θ power, increased with increasing age. However, the best-fitting coefficient for age at the temporoparietal sites was slightly negative, indicating an age-related decrease in temporoparietal α power. The significant coefficient for sex, indicating greater α power in females than males at the mean age of 42, was much greater than the effect of sex estimated for δ and θ power. Finally, the age and age^2^ coefficients were generally larger for females than males, indicating greater age-related changes in α power for females.

#### Beta power

**Figure 7.**
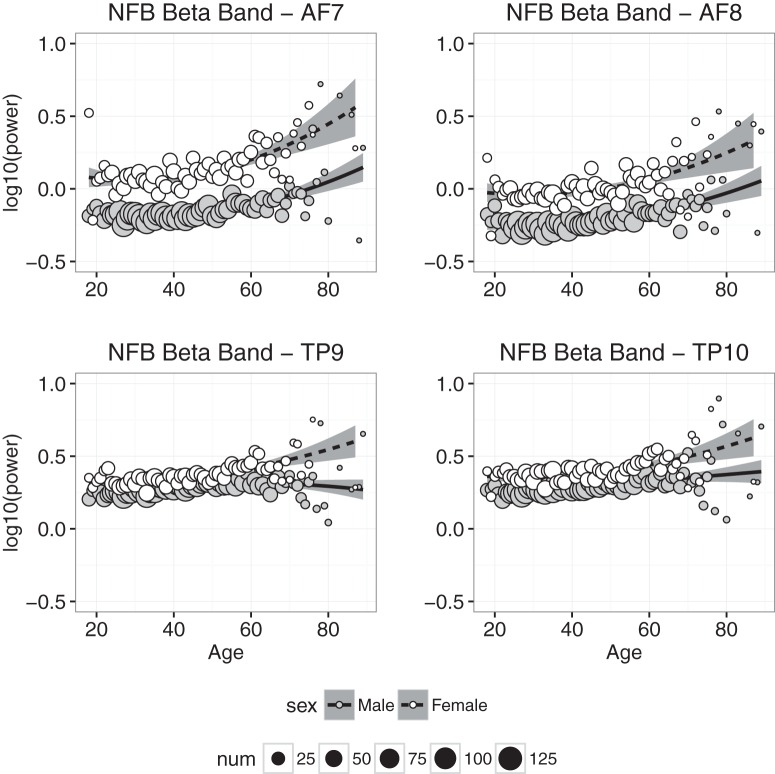
Beta band power plotted against age for males (gray symbols) and females (white symbols). Each point represents the mean for that age; symbol size represents how many individuals were used to compute the mean. Regression was used to compute the best-fitting curves separately for males and females, and the shaded regions represent 95% confidence intervals.

Beta power measured at each electrode in the NFB condition is plotted as a function of age in Fig. 7. As was found with α power, (1) there is clear evidence that β power measured at frontal electrodes increased with age; (2) β power was on average significantly higher in females than males; and (3) the sex difference and the trend across age were much smaller in data from temporoparietal electrodes, but unlike α, still highly significant (Figs. 6 and 7). The regression results in the CAL and NFB conditions generally were consistent with these observations—the coefficients for age, age^2^, and sex were significantly greater than zero—although the model accounted for >50% of the age-related variance in β power at frontal and temporoparietal electrodes.

#### Alpha peak frequency

**Figure 8.**
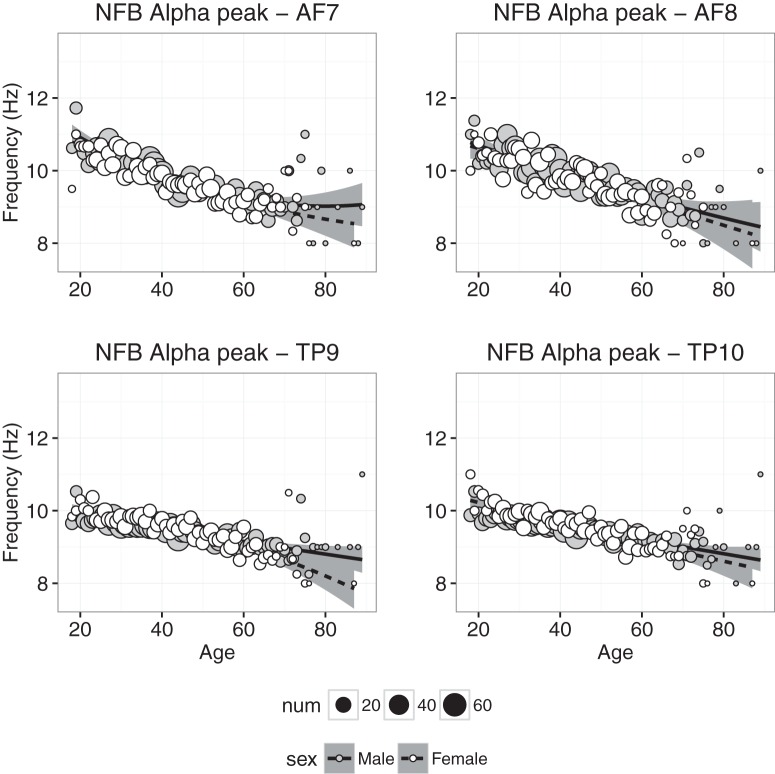
Alpha peak frequency plotted against age for males (gray symbols) and females (white symbols). Each point represents the mean for that age; symbol size represents how many individuals were used to compute the mean. Regression was used to compute the best-fitting curves separately for males and females, and the shaded regions represent 95% confidence intervals.

Alpha peak frequency in the NFB condition is plotted as a function age for each electrode in Fig. 8. First, the α peak frequency analysis differs from the other analyses because not all participants had a clear α peak frequency. In fact, of the 6029 participants, ∼88% had a peak frequency in the α range at the temporoparietal sites, while only 50% had an α peak frequency in the frontal sites. More specifically, at each channel, the following number of participants had α peak frequencies during the NFB session: TP9 (5374), TP10 (5379), AF7 (3085), and AF8 (2806). Similarly, the number of participants with α peak frequencies during the CAL session were as follows: TP9 (5136), TP10 (5320), AF7 (3111), and AF8 (2939; note: the weights in the WLS regression models were adjusted to reflect these numbers for the α peak frequency analyses). Importantly, α peak frequencies were found for categorically more of the temporoparietal sites compared with the frontal sites, which is consistent with the grand average power spectral density (Fig. 1) showing a clear peak in the α range for sites TP9 and TP10, but not for AF7 and AF8. Regardless, even after exclusion of observers without visible α peak frequencies, we had sufficient data to complete the analyses across the life span for each sex.

At all four electrodes, the α peak frequency exhibited a steady decline between 20 and 60 years of age. Compared with effects of age on the various power bands, age-related changes in α peak frequency exhibit a much smaller quadratic component and a much smaller difference between males and females. Regression analyses of the CAL and NFB data were consistent with these observations, though the model accounted for more age-related variance at all four electrodes in the NFB condition than the CAL condition. At temporoparietal sites, the trend across age was significantly more negative for females than males. Also note that the effect of age was slightly greater for α measured at frontal electrodes than temporoparietal electrodes (Tables 2 and 3).

### Alpha asymmetry

Alpha asymmetry reflects the difference between left and right α power, measured by subtracting the log_10_-transformed α power in the left hemisphere from the log_10_-transformed α power in the right hemisphere. The asymmetry is calculated separately at the frontal and temporoparietal sites; a negative asymmetry value reflects stronger left than right α power, and a positive asymmetry value reflects stronger right than left α power. Increased α power is typically associated with increased inhibition, and thus α power is thought to be inversely related to brain activity; increased α in one hemisphere is interpreted as decreased overall activity in that hemisphere. For example, a negative α asymmetry value typically is interpreted as showing greater neural activity in the right hemisphere relative to the left hemisphere.

**Figure 9.**
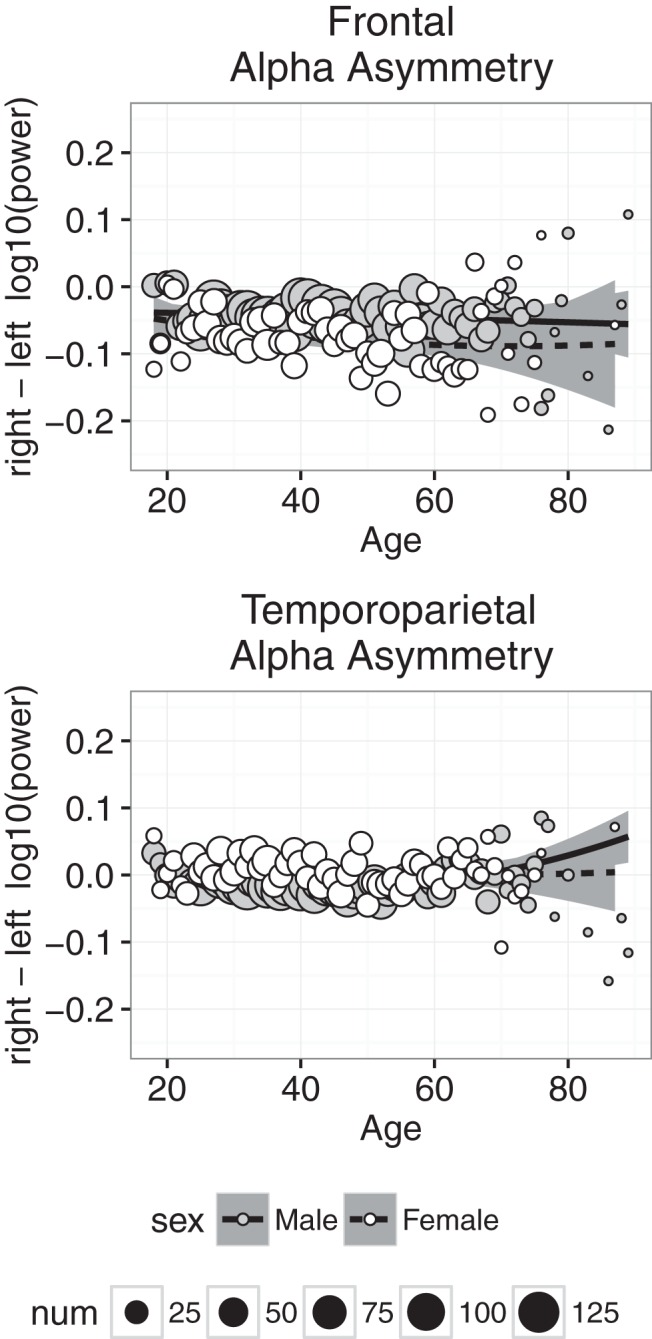
Alpha asymmetry measured at frontal (top) and temporoparietal (bottom) electrodes plotted against age for males (gray symbols) and females (white symbols). Each point represents the mean for that age; symbol size represents how many individuals were used to compute the mean. Regression was used to compute the best-fitting curves separately for males and females, and the shaded regions represent 95% confidence intervals.

Alpha asymmetry is plotted as a function of age in Fig. 9. At frontal electrodes, the asymmetry was slightly negative, indicating that α power was relatively greater in the right than left hemisphere, and the asymmetry was more negative in females than males. At temporoparietal electrodes, the average asymmetry was slightly positive or zero, and the asymmetry was slightly more positive in females than males. Finally, at both frontal and temporoparietal sites, we found little evidence for significant age-related changes in α asymmetry. The regression analyses were consistent with these observations: the best-fitting intercept was significantly less than zero at the frontal electrodes in the CAL and NFB conditions and significantly greater than zero at the temporoparietal electrodes in the CAL condition; in both conditions, the sex coefficient was significantly less than zero at frontal electrodes and significantly greater than zero at temporoparietal electrodes, and the effect of age was small in all conditions. Furthermore, in all cases, the model failed to account for at least 50% of the variance, again suggesting that there was very little systematic age-related variance in α asymmetry.

## Discussion

We collected frontal and temporoparietal EEG data from 6029 individuals ranging in age from 18 to 88 years while they performed a Category Exemplar Task and an MBSR-based exercise conducted at home using the Muse headband. We investigated how EEG power in the traditional frequency bands, α peak frequency, and α asymmetry changed as a function of age and sex. Our aim was to use the powerful sample size of the data collected using the Muse to characterize both large and subtle changes in EEG dynamics.

We found that EEG power was stronger in temporoparietal than frontal leads (Fig. 1). This finding was expected, given that all channels were referenced to Fpz, although temporoparietal regional power is generally higher than frontal regions (Dustman et al., 1993; Coben et al., 2008). Our findings highlight the prevalence of a sex difference in the general population, with females having higher overall EEG power in most frequency bands (Veldhuizen et al., 1993). The sex differences are consistent with previous studies demonstrating higher power in females in δ and α bands during sleep (Latta et al., 2005), slow waves during sleep (Mourtazaev et al., 1995), overall β activity (Mundy-Castle, 1951), and δ, θ, α, and β bands during rest and photic stimulation (Wada et al., 1994; Carrier et al., 2001). These replications of previously reported studies suggest that valid and reliable aspects of EEG can be measured when Muse is used by consumers in an uncontrolled environment. Overall higher power in female EEG may be related to various functional and anatomical sex differences, including thicker cortical gray matter in females (Sowell et al., 2007), increased neuronal processes in females (Rabinowicz et al., 1999), and different skull thicknesses (Roche, 1953; Hagemann et al., 2008).

Power in the slow wave δ and θ bands decreased significantly with age (Figs. 4 and 5), and although the decrease was slight, it is consistent with the downward trend of these slow waves observed during childhood (Matthis et al., 1980; Benninger et al., 1984; Marshall et al., 2002; Otero et al., 2003). The downward trend in δ and θ is accompanied by increased power in the α and β bands (Fig. 6 & 7), which has not been previously reported, but is consistent with trends observed throughout childhood (Benninger et al., 1984; Carrier et al., 2001).

Consistent with previous findings, β power increased significantly with age and was greater in females than males (Mundy-Castle, 1951; Carrier et al., 2001). Although our methods were not designed to measure β activation in response to stimulus/task demands, increased β power in older adults may be consistent with work demonstrating an association between poor attention and β modulation (Gola et al., 2012). Increased baseline β activity may be associated with less β modulation overall: training with β neurofeedback is associated with increased attention and arousal, which is thought to explain both lower reaction times and improved sensitivity in a sustained attention task (Egner and Gruzelier, 2004). Sustained visual attention has also been linked to β activity (Wróbel, 2000), underscoring the importance of understanding how β activity changes with age, and whether these changes are associated with age-related changes in attention. The link between β modulation and baseline β activity is not yet established, but the strong age-related trend observed here suggests it may merit further investigation.

Females had significantly greater frontal α power than males, consistent with previous results (Latta et al., 2005). As indicated by the intercepts of the linear models, frontal α power was greater during CAL than NFB, suggesting a task-mediated modulation. Alpha power is known to be modulated by task demands (Payne et al., 2013), fatigue (Crabbe and Dishman, 2004), and mindfulness meditation (Kerr et al., 2011), all of which are likely at play during use of the Muse.

The strongest age-related change we saw in the data was a year-by-year slowing of the α peak frequency (Fig. 8), which decreased similarly for males and females. This decrease was strongest at frontal sites. The slowing of α replicates extensive research demonstrating that α peak frequency is age dependent (Woodruff and Kramer, 1979; Duffy et al., 1984; Giaquinto and Nolfe, 1986; Clark et al., 2004). Alpha slowing throughout adulthood is in contrast to the increase in the α peak frequency during normal childhood development (Marshall et al., 2002). The shift at the two ends of the lifespan do not seem to be perfectly symmetrical, with changes in the adult years being very gradual compared with rapid changes throughout childhood. The age-related decline of the α peak frequency may be associated with reduced working memory capability (Clark et al., 2004). Using neurofeedback, Angelakis et al. (2007) demonstrated that training older adults to increase their peak α frequency was positively correlated with cognitive processing speed and executive function, but not with improved memory. It is also worth saying that correlations between α peak frequency and cognitive measures should consider the role of β, given our earlier discussion of β power being associated with attentional control. In our sample, there was a significant negative correlation between α peak frequency and β power, where β power increased as α peak frequency slowed (AF7: *r* = –0.34, β = –0.084, *p* < 0.0002, with similar results at other channels). This relation may be important, because changes in β power and α peak have both been independently associated with cognitive/attentional deficits, but further direct investigation is required.

We used frontal α asymmetry as a proxy to measure differences in relative left/right EEG activity. Participants, especially females, presented with negative frontal asymmetry during both sessions, representing greater relative right frontal activity (Davidson et al., 2000). Relatively greater right frontal activity is associated with the behavioral inhibition system (compare the behavioral activation system, together known as BIS/BAS), which entails a general tendency to withdraw and disengage from aversive stimuli and a greater propensity to experience negative emotion (Sutton and Davidson, 1997), although this relation has been questioned (Coan and Allen, 2003). Also, frontal asymmetry was very similar during CAL and NFB sessions, suggesting that it is likely trait and not state dependent (Tomarken et al., 1992; Mathewson et al., 2015). Further analyses are required to test the stability/test-retest reliability of asymmetry during sessions with the Muse.

If asymmetry is a valid index of affective types, then the overall negative asymmetry is especially interesting given our data: participants were consumers using a neurofeedback device to assist in mindfulness-based exercises at home. Besides early adopters likely comprising a significant portion of the current consumers (who comprise more men than women in markets such as the United States; Chau and Lung Hui, 1998; Ipsos, 2012), there ought to be a sizeable proportion of consumers who used the Muse specifically to improve their mental well-being. Therefore, we can expect the user-base to present with negative affect/negative asymmetry, especially given that we restricted our sample to the first five sessions per participant. InteraXon’s constantly growing, updated database should be used to compare the same users after extensive meditation sessions. In fact, MBSR training with healthy older individuals has been linked to improved well-being and a reduced rightward shift in activity (Moynihan et al., 2013). Interestingly, their results suggest a normal, age-related rightward trajectory of asymmetry, with MBSR helping prevent/reduce this trajectory, which is then associated with improved well-being on several fronts, including executive and immune functions (Davidson et al. (2003).

There is growing evidence linking α asymmetry and mindfulness, and mindfulness to enhanced physical and mental well-being. For example, mindfulness exercises can modulate somatosensory attention (Kerr et al., 2013), consistent with the view that mindfulness enhances attention to bodily sensations (Kabat-Zinn, 1994; Kerr et al., 2013). More generally, mindfulness is associated with attention regulation (Rani and Rao, 1996; Tang et al., 2007), which is tightly linked to α oscillations (Payne and Sekuler, 2014), suggesting that α training through mindfulness may be beneficial for enhancing attentional control. Other benefits of mindfulness-based exercises include reduced emotional interference (Ortner et al., 2007) and increased regulation (Arch and Craske, 2006), lower perceived stress and increased positive affect (Tang et al., 2007; Carmody and Baer, 2008; Nyklíček and Kuijpers, 2008), reduced fatigue and anxiety (Zeidan et al., 2010), and improvements in working memory and processing fluency (Chambers et al., 2008; Zeidan et al., 2010). Future replications of EEG patterns measured in laboratory settings with data collected in the home with Muse will help us to generalize experimental results to real-world scenarios and better understand the physical and psychological benefits of mindfulness-related exercises.

In conjunction with the above discussion, it is worthwhile to be cognizant of the nature of the data and any possible issues of selection bias (Hernán et al., 2004). Although these issues are unlikely to impact our results in any significant way due to the massive sample size, the consumer product may have attracted individuals seeking to begin, or continue, meditation exercises. As such, the data presented here may not be entirely representative of the normal population, but rather a population of meditative individuals, or a population of individuals who share some trait that makes them more likely to be interested in meditation. The data presented here were not tagged with information regarding the users’ intents and experiences with mediation; however, our understanding is that InteraXon has begun to collect this data as part of a software update, allowing future researchers to address any potential issue of bias in participant selection in an updated and much larger database. Furthermore, the fact that our pattern of results is consistent with previous results found in smaller, but well-controlled, studies increases our confidence that selection bias effects did not drive our results. As such, we focus our conclusions on the true power of this study: the enormous sample size with data points at every adult age, separately for males and females.

Overall, with increasing age there was a shift in EEG power toward higher frequency bands at the expense of the lower frequencies. Peak α frequency underwent a year-by-year slowing, and Muse users, especially females, exhibited relatively greater right frontal activity. We demonstrated large-scale replication of previous small-scale laboratory studies, which we see as a validation of not only these previous studies, but also the Muse database, highlighting the utility of doing further, more intricate analyses using this large and perhaps more representative community-based participant database. Our primary aim was to demonstrate the utility of using such datasets to look at EEG dynamics at the population level, as they provide remarkable power to detect sex differences and gradual changes with age.

## Synthesis

Reviewing Editor: Philippe Tobler, University of Zurich Decisions are customarily a result of the Reviewing Editor and the peer reviewers coming together and discussing their recommendations until a consensus is reached. When revisions are invited, a fact-based synthesis statement explaining their decision and outlining what is needed to prepare a revision will be listed below. The following reviewer(s) agreed to reveal their identity: Simon Hanslmayr.

The authors present a study exploring age-related changes of EEG oscillation patterns in a large cross-sectional community sample of adults ages 18 to 88 years practicing meditation under guidance of a commercial biofeedback EEG system during a cognitive task with eyes closed used as calibration (CAL) and a meditation session under biofeedback guidance (NFB) The study has much strength, particularly due to the large sample size, but this came at some cost, particularly since the data acquisition was consequently not so well controlled, both compared to smaller studies conducted in EEG-Labs. Thus, the authors largely confirmed with the current dataset what was previously known from smaller, but better controlled studies regarding ageing effects and sex differences.

The authors addressed most of the concerns related to the previous version.

However, several issues remain.

The authors are not able to spell out their analysis protocols (i.e. preprocessing or checks for signal quality). They argue that the details for these algorithms are subject to trade secrets, which is of course fair enough, but this conflicts with the scientific principles of the peer review process and makes it impossible to evaluate these aspects of the paper. In the minimum, the authors need to provide information about where and how the algorithms have been validated in published form, otherwise the paper cannot be published in eNeuro.

Alternatively, the data could be analyzed with non-proprietary software and authors could check the included datasets for EMG and ECG artefacts independently of the closed algorithms applied by the InteraCon Muse system/database.

Also, please add information about the type of machine learning applied to identify eye-blinks and EMG activity - the latter may also be checked using power in higher frequency bands in the included data).

Moreover, the sample may be highly selective (individuals using MUSE in the given timeframe), so some check and if possible adjustment for selection bias may further strengthen the paper. E.g. the inclusion of adults practicing meditation using biofeedback guidance may not be representative, suggesting a volunteer bias. In addition, the datasets from 6029 included participants come from a larger database, so the authors may discuss and assess possible selection or missing data bias due to e.g. adherence or the artefact criteria applied. The authors may refer to Hernan (2004) "A Structural Approach to Selection Bias" for further discussion of these issues.

Finally, please clarify whether NFB was performed with eyes closed.
